# Transplant hepatology and diversity: A decade‐long analysis (2013–2022)

**DOI:** 10.1002/jgh3.13048

**Published:** 2024-02-27

**Authors:** Abdulmalik Saleem, Mahfujul Z Haque, Saif Affas, Maaz Munawar, Syed‐Mohammed Jafri

**Affiliations:** ^1^ Department of Internal Medicine Henry Ford Hospital Detroit Michigan USA; ^2^ Michigan State University College of Human Medicine East Lansing Michigan USA; ^3^ Department of Internal Medicine Ascension Providence Southfield Michigan USA; ^4^ University of Michigan, College of Science Ann Arbor Michigan USA; ^5^ Department of Hepatology Henry Ford Health Detroit Michigan USA

**Keywords:** graduate medical education, training, transplant hepatology, underrepresented minorities

## Abstract

Diversity among physicians has been shown to positively impact patient care. Physicians from minority backgrounds are more likely to serve underserved communities and be involved in health disparities research. Efforts to increase the proportion of underrepresented minorities and women in medicine will help prepare a physician workforce that best cares for a diversifying nation. The purpose of this paper was to highlight trends in sex and ethnic representation among incoming U.S. transplant hepatology trainees over a 10‐year period.

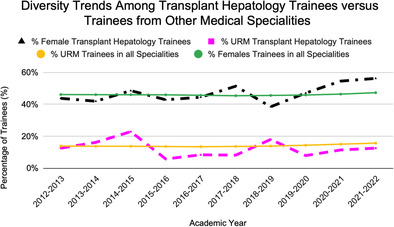

## Introduction

The U.S. Census Bureau projects that by 2044, more than half of all Americans will belong to a minority group.^1^ As the proportion of minorities continues to rise, a diverse physician workforce becomes increasingly important. Diversity among physicians has been shown to positively impact patient care.^2^ Physicians from minority backgrounds are more likely to serve underserved communities and be involved in health disparities research.^3^ Efforts to increase the proportion of underrepresented minorities (URM) in medicine will help prepare a physician workforce that best represents and cares for our diversifying nation.

The purpose of this paper was to highlight trends in sex and ethnic representation among incoming U.S. transplant hepatology (TH) trainees over a 10‐year period.

## Methods

We utilized the annual Graduate Medical Education (GME) Census to gather and analyze data on the sex and ethnic identities of matriculating TH trainees.^5^ Microsoft Excel was used to compare trends in female and URM representation among trainees over a 10‐year period from 2013 to 2022.

Trainees self‐identified their sex as female or male and their ethnicity as Asian, Black, Hispanic, American Indian or Alaskan Native, Native Hawaiian or Pacific Islander, White, Other or Unknown, or Multiracial on the census. URM were defined as fellows who self‐identified as Black, Hispanic, American Indian or Alaskan Native, or Native Hawaiian or Pacific Islander.

## Results

**Figure 1:**
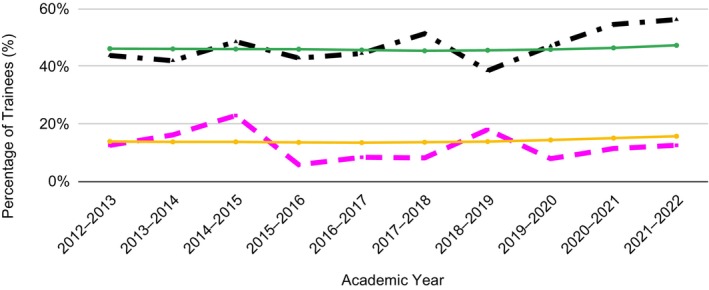
Diversity trends among transplant hepatology trainees *versus* trainees from other medical specialties. 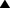, % female transplant hepatology trainees; 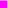, % underrepresented minorities (URM) transplant hepatology trainees; 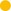, % URM trainees in all specialities; 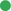, % females trainees in all specialities.

Figure 1 displays the sex and ethnic trends among TH fellows in comparison with trainees from all specialties. A total of 184 trainees were woman, among 388 total trainees. Thus, women represented 47.42% of all trainees. The proportion of TH fellows who identified as female was 43.75% in 2013 and 56.25% in 2022, compared with 46.12% and 47.29% for all other specialties. Forty‐seven trainees over this period were URM of 388 total trainees. This represented 12.11% of all trainees. The proportion of TH fellows who identified as URMs was 12.50% in 2013 and 12.50% in 2022, compared with 13.82% and 15.64% for all other specialties (Appendix S1, Supporting information).

## Discussion

Our study assessed the changes in the percentage of URM and females in TH fellows from 2013 to 2022. Over a 10‐year span, approximately 12% of all trainees were URM and 47% of all trainees were women. Our analysis reveals URM representation among TH fellows that is consistently lower than URM representation among all other specialties over this 10‐year period.^2^, ^3^, ^5^ Female representation among TH fellows has a more variable pattern, with a recent increase in female representation among hepatology trainees compared with trainees from all specialties.

There is a continued need to implement programs that promote diversity among TH fellowship programs as well as all other medical specialties. Especially in gastroenterology (GI), understanding differences in nutrition and diet among individuals from different backgrounds are critical.^4^ It is unclear why there is a lower representation of URM among TH fellows; however, previous studies in other specialties have concluded that a lack of mentorship and early specialty exposure significantly contributes to this.^6^ Having trainees of diverse backgrounds may contribute to reducing disparities in care. This can be accomplished by having strong accessible mentorship for those who come from backgrounds historically underrepresented in medicine. Limitations include limited sample size, limited time frame of the study (2013–2022), and programs that do not participate in the GME census.

The underrepresentation of URM in TH has far‐reaching clinical implications, including disparities in research, patient care, access to transplantation, and the development of effective management strategies for liver diseases. Addressing these disparities is crucial for promoting equity and inclusivity in hepatology and liver transplantation, ultimately contributing to improved patient outcomes and a more diverse and representative healthcare workforce. We hope that this paper can play a fundamental role in sparking future conversations about steps we can take to influence diversity shifts in the field.

## Supporting information


**Appendix S1.** Supporting information.
