# Supplementary figures and images for: Transplant hepatology and diversity: A decade‐long analysis (2013–2022)
Source: JGH Open. 2024 Feb 27;8(2):e13048. doi: 10.1002/jgh3.13048 (PMC10898196; doi:10.1002/jgh3.13048)

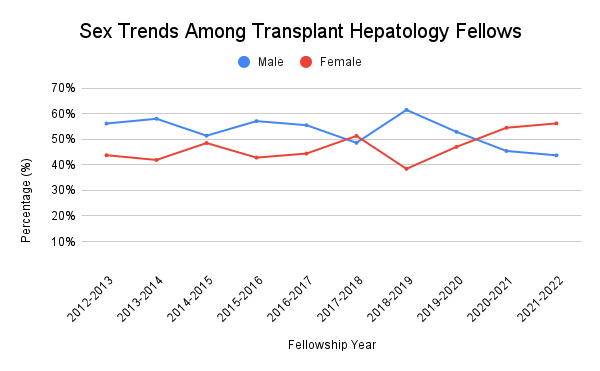


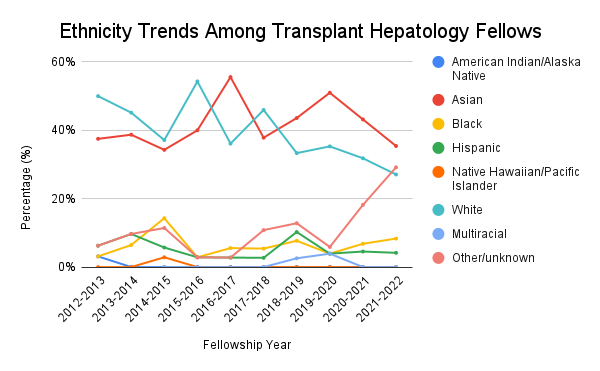


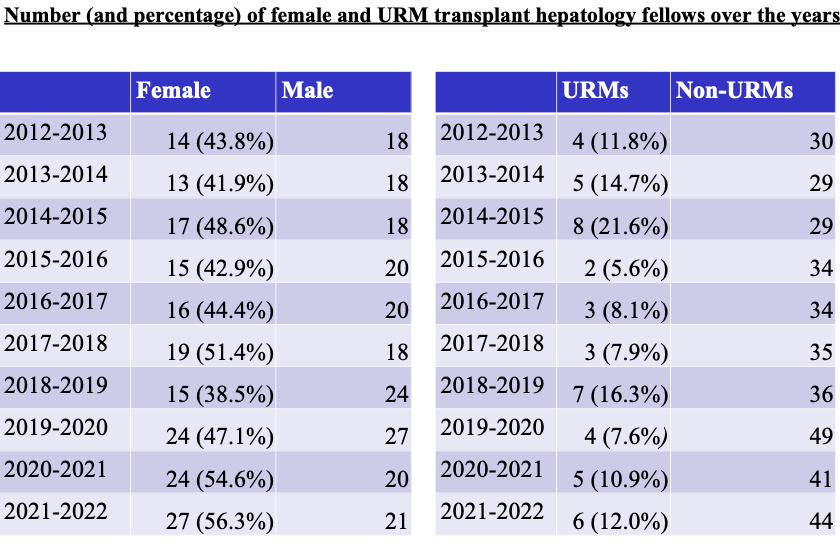

Supplement: Supplementary file 1 — Appendix S1. Supporting information. [file JGH3-8-e13048-s001.docx]
